# Synthesis, photophysical and electrochemical properties of pyridine, pyrazine and triazine-based (D–π–)_2_A fluorescent dyes

**DOI:** 10.3762/bjoc.15.167

**Published:** 2019-07-22

**Authors:** Keiichi Imato, Toshiaki Enoki, Koji Uenaka, Yousuke Ooyama

**Affiliations:** 1 Department of Applied Chemistry, Graduate School of Engineering, Hiroshima University, 1-4-1 Kagamiyama, Higashi-Hiroshima 739-8527, Japan

**Keywords:** D–π–A structure, fluorescent dyes, pyrazine, pyridine, triazine

## Abstract

The donor–acceptor–π-conjugated (D–π–)_2_A fluorescent dyes **OUY**-**2**, **OUK-2** and **OUJ-2** with two (diphenylamino)carbazole thiophene units as D (electron-donating group)–π (π-conjugated bridge) moiety and a pyridine, pyrazine or triazine ring as electron-withdrawing group (electron-accepting group, A) have been designed and synthesized. The photophysical and electrochemical properties of the three dyes were investigated by photoabsorption and fluorescence spectroscopy, Lippert–Mataga plots, cyclic voltammetry and density functional theory calculations. The photoabsorption maximum (λ_max,abs_) and the fluorescence maximum (λ_max,fl_) for the intramolecular charge-transfer characteristic band of the (D–π–)_2_A fluorescent dyes show bathochromic shifts in the order of **OUY-2** < **OUK-2** < **OUJ-2**. Moreover, the photoabsorption bands of the (D–π–)_2_A fluorescent dyes are nearly independent of solvent polarity, while the fluorescence bands showed bathochromic shifts with increasing solvent polarity (i.e., positive fluorescence solvatochromism). The Lippert–Mataga plots for **OUY**-**2**, **OUK-2** and **OUJ-2** indicate that the Δμ (= μ_e_ − μ_g_) value, which is the difference in the dipole moment of the dye between the excited (μ_e_) and the ground (μ_g_) states, increases in the order of **OUY-2** < **OUK-2** < **OUJ-2**. Therefore, the fact explains our findings that **OUJ-2** shows large bathochromic shifts of the fluorescence maxima in polar solvents, as well as the Stokes shift values of **OUJ-2** in polar solvents are much larger than those in nonpolar solvents. The cyclic voltammetry of **OUY**-**2**, **OUK-2** and **OUJ-2** demonstrated that there is little difference in the HOMO energy level among the three dyes, but the LUMO energy levels decrease in the order of **OUY-2** > **OUK**-**2** > **OUJ**-**2**. Consequently, this work reveals that for the (D–π–)_2_A fluorescent dyes **OUY**-**2**, **OUK-2** and **OUJ-2** the bathochromic shifts of λ_max,abs_ and λ_max,fl_ and the lowering of the LUMO energy level are dependent on the electron-withdrawing ability of the azine ring, which increases in the order of **OUY-2** < **OUK-2** < **OUJ-2**.

## Introduction

Donor–π-conjugated–acceptor (D–π–A) dyes are constructed of an electron-donating group (D) such as a diphenyl or dialkylamino group and an electron-withdrawing group (electron-accepting group, A) such as a nitro, cyano, and carboxy group or an azine ring such as pyridine, pyrazine and triazine linked by π-conjugated bridges such as oligoenes and heterocycles. Thus, the D–π–A dyes exhibit intense photoabsorption and fluorescence emission properties based on the intramolecular charge transfer (ICT) excitation from the D moiety to the A moiety [1–4]. Moreover, the D–π–A structure possesses considerable structural characteristics: the increase in the electron-donating and electron-accepting abilities of the D and A moieties and the expansion of π conjugation, respectively, can lead to a decrease in the energy gap between the HOMO and LUMO because the highest occupied molecular orbital (HOMO) is localized over the π-conjugated system containing the D moiety, and the lowest unoccupied molecular orbital (LUMO) is localized over the A moiety. Thus, the photophysical and electrochemical properties based on the ICT characteristics of D–π–A dyes should be tuneable by not only the electron-donating ability of D and the electron-accepting ability of A, but also by the electronic characteristics of the π bridge. Consequently, the D–π–A dyes are of considerable practical concern as a useful fluorescence sensor for cation, anion and neural species [5–14], an efficient emitter for organic light emitting diodes (OLEDs) [15–24], and a promising photosensitizer for dye-sensitized solar cells (DSSCs) [25–34].

**Figure 1:**
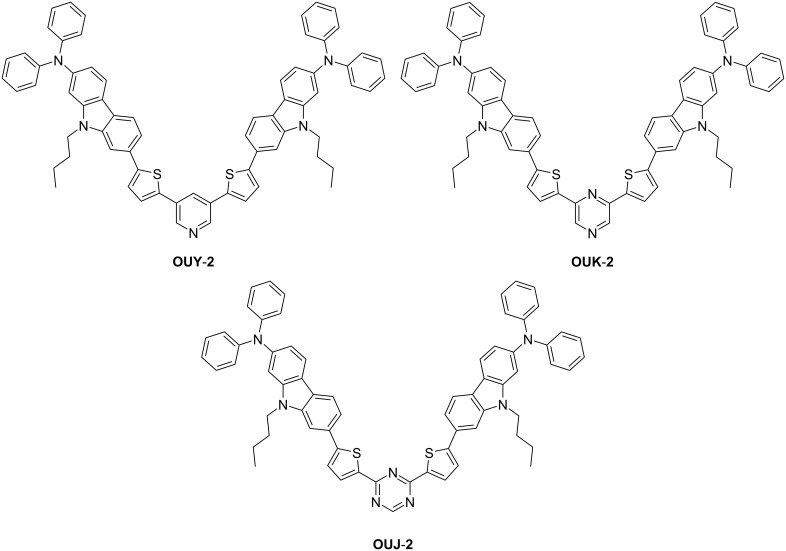
Chemical structures of the (D–π–)_2_A fluorescent dyes **OUY-2**, **OUK-2** and **OUJ-2**.

Thus, in this work, to gain insight into the photophysical and electrochemical properties of D–π–A fluorescent dyes with an azine ring as electron-withdrawing group, we have designed and synthesized the (D–π–)_2_A fluorescent dyes **OUY**-**2**, **OUK-2** and **OUJ-2** with two (diphenylamino)carbazole thiophene units as the D–π moiety and a pyridine, pyrazine or triazine ring as the A moiety (Figure 1), although we have already reported the synthesis of (D–π–)_2_A fluorescent dyes **OUY-2** [2] and **OUK-2** [3–4] and their partial photopysical and electrochemical properties. One advantage of (D–π–)_2_A fluorescent dyes over other D–π–A fluorescent dyes is their broad and intense photoabsorption spectral features. Herein, based on photoabsorption and fluorescence spectroscopy, Lippert–Mataga plots, cyclic voltammetry and density functional theory (DFT) calculations, we reveal the photophysical and electrochemical properties of the (D–π–)_2_A fluorescent dyes **OUY**-**2**, **OUK-2** and **OUJ-2**.

## Results and Discussion

### Synthesis

**Scheme 1:**
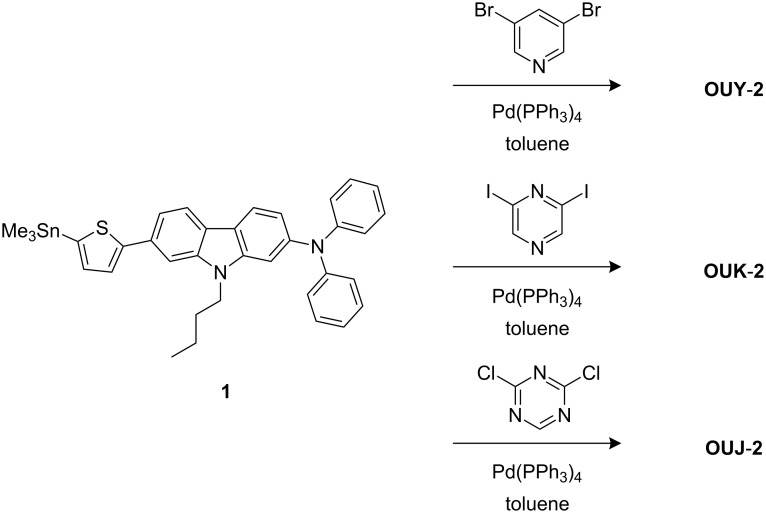
Synthesis of **OUY-2**, **OUK-2** and **OUJ-2**.

The (D–π–)_2_A fluorescent dyes **OUY-2** [2], **OUK-2** [3–4] and **OUJ-2** were prepared by Stille coupling of stannyl compound **1** [3] with 3,5-dibromopyridine, 2,6-diiodopyrazine, and 2,4-dichloro-1,3,5-triazine, respectively (Scheme 1; see Experimental section for the synthetic procedure of **OUJ-2**).

### Optical properties

**Figure 2:**
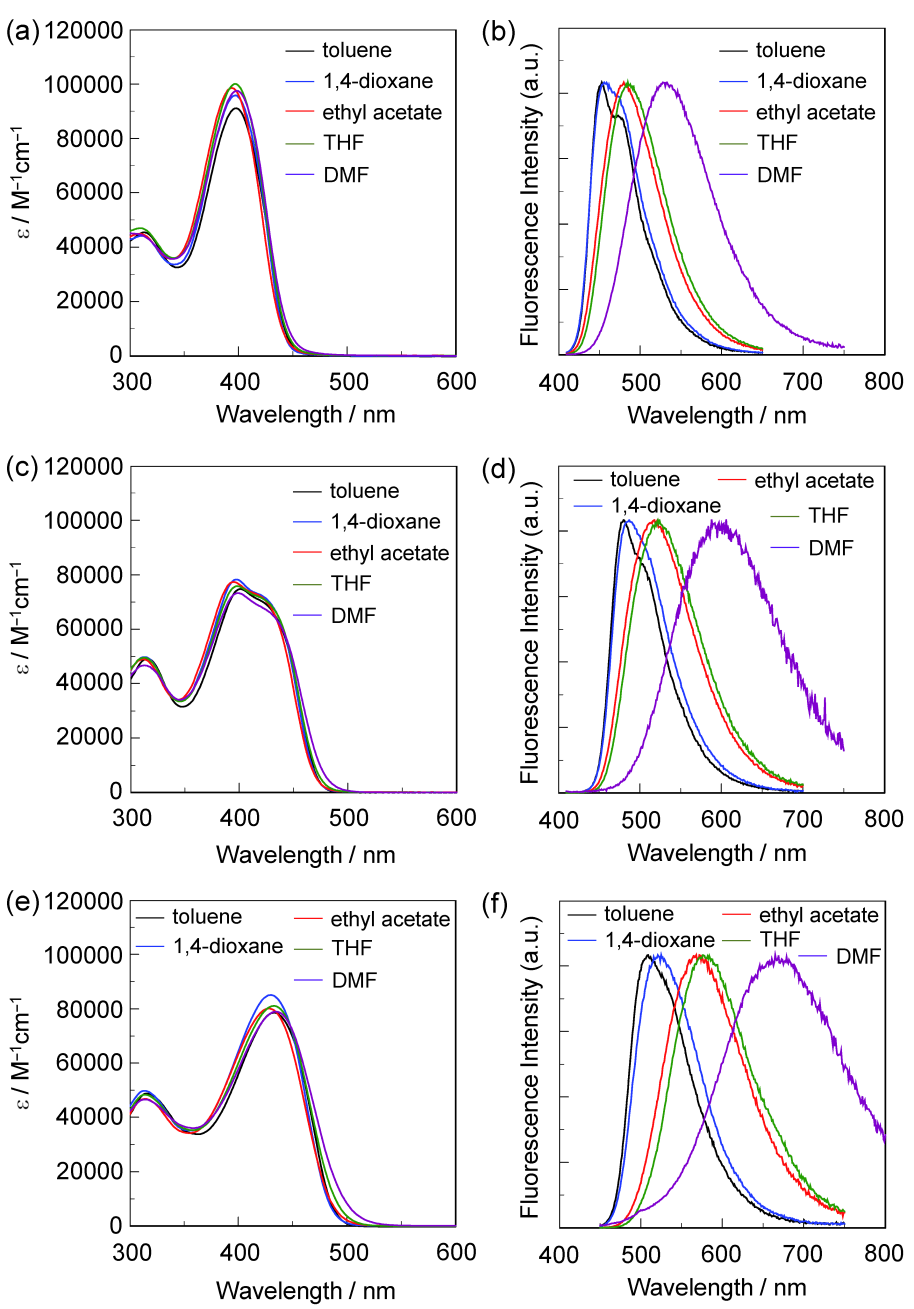
(a) Photoabsorption and (b) fluorescence (λ^ex^ = ca. 400 nm) spectra of **OUY-2** in various solvents. (c) Photoabsorption and (d) fluorescence (λ^ex^ = ca. 400 nm) spectra of **OUK-2** in various solvents. (e) Photoabsorption and (f) fluorescence (λ^ex^ = ca. 430 nm) spectra of **OUJ-2** in various solvents.

The photoabsorption and fluorescence spectra of **OUY**-**2**, **OUK-2** and **OUJ-2** in various solvents are shown in Figure 2, and their optical data are summarized in Table 1. **OUY**-**2**, **OUK-2** and **OUJ-2** in toluene as a non-polar solvent show the photoabsorption maximum (λ_max,abs_) at 398 nm, 401 nm and 433 nm, respectively, which is assigned to the ICT excitation from the two (diphenylamino)carbazole thiophene units as D–π moiety to a pyridine, pyrazine or triazine ring as A moiety. For **OUK-2**, the shoulder band was observed at around 430 nm. Thus, the ICT-based photoabsorption band of the three dyes appears at a longer wavelength region in the order of **OUY-2** < **OUK-2** < **OUJ-2**, which is in agreement with the increase in the electron-withdrawing ability of the azine ring in the order of pyridyl group < pyrazyl group < triazyl group. The photoabsorption spectra of the three dyes are nearly independent of solvent polarity. This indicates that the electronic and structural characteristics of both the ground and Franck–Condon (FC) excited states do not differ much with a change in solvent polarity. The molar extinction coefficient (ε_max_) for the ICT band is ca. 100000 M^−1^ cm^−1^ for **OUY-2**, 75000 M^−1^ cm^−1^ for **OUK-2** and 80000 M^−1^ cm^−1^ for **OUJ-2**. The corresponding fluorescence maximum (λ_max,fl_) of the three dyes in toluene also appears at a longer wavelength region in the order of **OUY-2** (453 nm) < **OUK-2** (480 nm) < **OUJ-2** (509 nm). Interestingly, in contrast to the photoabsorption spectra, the fluorescence spectra are strongly dependent on the solvent polarity, that is, the three dyes showed a bathochromic shift of the fluorescence band with increasing solvent polarity from toluene to DMF (i.e., positive fluorescence solvatochromism). Thus, the Stokes shift (SS) values of the three dyes increase with increasing solvent polarity. Compared with **OUY**-**2**, **OUK-2** and **OUJ-2** exhibit significant fluorescence solvatochromic properties, that is, the two dyes show a significant decrease in the fluorescence quantum yield (Φ_f_) in a polar solvent such as DMF (Φ_f_ = 0.59, 0.14 and 0.09 for **OUY**-**2**, **OUK-2** and **OUJ-2**, respectively), although in relatively low polar solvents **OUK-2** and **OUJ-2** exhibit a higher Φ_f_ value (0.48–0.65 and 0.72–0.86, respectively) than **OUY**-**2** (Φ_f_ = 0.38–0.58). For **OUK-2** and **OUJ-2**, the large bathochromic shifts of the fluorescence band with a significant decrease in the Φ_f_ value in polar solvents such as DMF might be arising from the twisted intramolecular charge transfer (TICT) excited state due to the twisting between the pyrazyl or triazyl group and the (diphenylamino)carbazole thiophene moiety, leading to non-radiative deactivation [1]. On the other hand, it is worth mentioning here that the brightness values (ε × Φ_f_) for **OUY-2**, **OUK-2** and **OUJ-2** in various solvents are fairly large (Table 1). Thus, the fact indicates that the (D–π–)_2_A fluorescent dyes have advantageous characteristics as emitters for OLEDs and fluorescence probes for biological imaging.

**Table 1:** Optical data of **OUY-2**, **OUK-2** and **OUJ-2** in various solvents.

Dye	Solvent	λ_max,abs_ [nm] (ε [M**^−^**^1^cm**^−^**^1^])	λ_max,fl_ [nm] (Φ_f_)^a^	Brightness [M**^−^**^1^cm**^−^**^1^]	Stokes shift [cm**^−^**^1^]

**OUY-2**	toluene 1,4-dioxane ethyl acetate THF DMF	398 (91100) 398 (95800) 394 (98500) 397 (100000) 399 (97500)	453 (0.38) 455 (0.40) 480 (0.39) 485 (0.58) 533 (0.59)	34600 38300 38400 58000 57500	3050 3147 4547 4570 6300
**OUK-2**	toluene 1,4-dioxane ethyl acetate THF DMF	401 (74800) 397 (78300) 398 (75800) 394 (77400) 399 (73300)	480 (0.48) 487 (0.62) 518 (0.55) 524 (0.65) 588 (0.14)	35900 48500 41700 50300 10200	4104 4655 5820 6296 8055
**OUJ-2**	toluene 1,4-dioxane ethyl acetate THF DMF	433 (78500) 430 (85100) 428 (80100) 433 (81100) 435 (78900)	509 (0.81) 525 (0.86) 568 (0.72) 576 (0.72) 665 (0.09)	63600 73200 57700 58400 7100	3448 4208 5758 5733 7950

^a^Fluorescence quantum yields (Φ_f_) were determined by using a calibrated integrating sphere system (λ^ex^ = 400 nm for **OUY-2**, 400 nm for **OUK-2**, and 430 nm for **OUJ-2**, respectively).

It is well accepted that the dipole–dipole interactions between the fluorescent dye and the solvent molecules are responsible for the solvent-dependent shifts in the fluorescence maxima [35–43]. Therefore, in order to understand the fluorescence solvatochromisms of **OUY**-**2**, **OUK-2** and **OUJ-2**, we have investigated the relationships between the solvent polarity-dependent shift of the fluorescence maximum and the dipole moment of dye molecule on the basis of the Lippert–Mataga equation (Equation 1) [44–46]:

[1] $$v_{st}=\frac{1}{4\pi \varepsilon_{0}}\cdot \frac{2\Delta \mu^{2}}{hca^{3}}\Delta f+const.$$

where

[2] $$\Delta f=\frac{\varepsilon -1}{2\varepsilon +1}-\frac{n^{2}-1}{2n^{2}+1}$$

**Figure 3:**
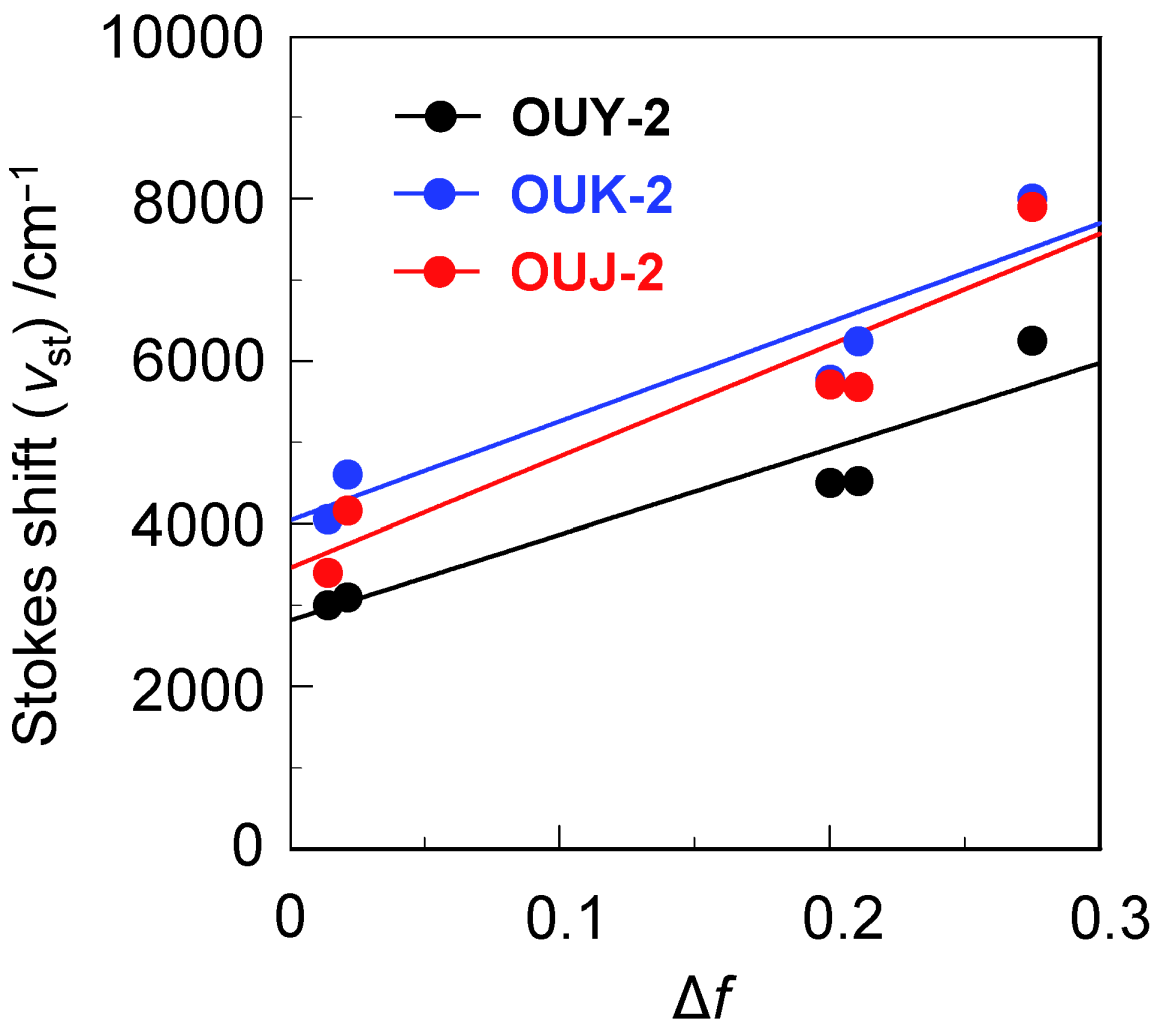
Correlation of the Stokes shift (*ν*_st_) and the orientation polarizability (Δ*f*) according to Equation 1 and Equation 2, respectively, for **OUY-2**, **OUK-2** and **OUJ-2**; solvent (*ε*, *n*, Δ*f*): toluene (2.38, 1.4969, 0.0132), 1,4-dioxane (2.21, 1.4224, 0.0205), ethyl acetate (6.02, 1.3724, 0.199), THF (7.58, 1.4072, 0.2096) and DMF (36.71, 1.4305, 0.274) [4].

Consequently, on the basis of Equation (1) and Equation (2), the change in the dipole moment, Δμ = μ_e_ − μ_g_, between the ground (μ_g_) and the excited (μ_e_) states can easily be evaluated from the slope of a plot of *ν*_st_ against Δ*f* (the Lippert–Mataga plot), where *ν*_st_ is the Stokes shift (Table 1), ε_0_ is the vacuum permittivity, *h* is Planck’s constant, *c* is the velocity of light, *a* is the Onsager radius of the dye molecule (7.81 Å, 7.99 Å and 7.91 Å for **OUY**-**2**, **OUK-2** and **OUJ-2**, respectively, estimated from DFT calculation at the B3LYP/6-31G(d,p) level of theory [47]), Δ*f* is the orientation polarizability, ε is the static dielectric constant, and *n* is the refractive index of the solvent. The Lippert–Mataga plots (Figure 3) for the three dyes show high linearity, indicating that for the three dyes the solvent-dependent shift in the fluorescence maximum is mainly attributed to the dipole–dipole interactions between the dye molecule and the solvent molecule. The slopes (*m*_sl_) became steep in the order of **OUY-2** (10500 cm^−1^) < **OUK-2** (12200 cm^−1^) < **OUJ-2** (13700 cm^−1^). The correlation coefficient (*R^2^*) value for the calibration curve regarding the three dyes is 0.90 for **OUY**-**2**, 0.88 for **OUK**-**2**, and 0.89 for **OUJ**-**2**, which indicates good linearity. The Δμ values increase in the order of **OUY-2** (22 D) < **OUK-2** (25 D) < **OUJ-2** (26 D), which corresponds to the increase in the electron-withdrawing ability of the azine rings (pyridyl group < pyrazyl group < triazyl group). Consequently, the Lippert–Mataga plots explains our findings that **OUJ-2** shows large bathochromic shifts in its fluorescence maximum in polar solvents, as well as the SS values for **OUJ-2** in polar solvents are much larger than those in nonpolar solvents (Table 1).

**Figure 4:**
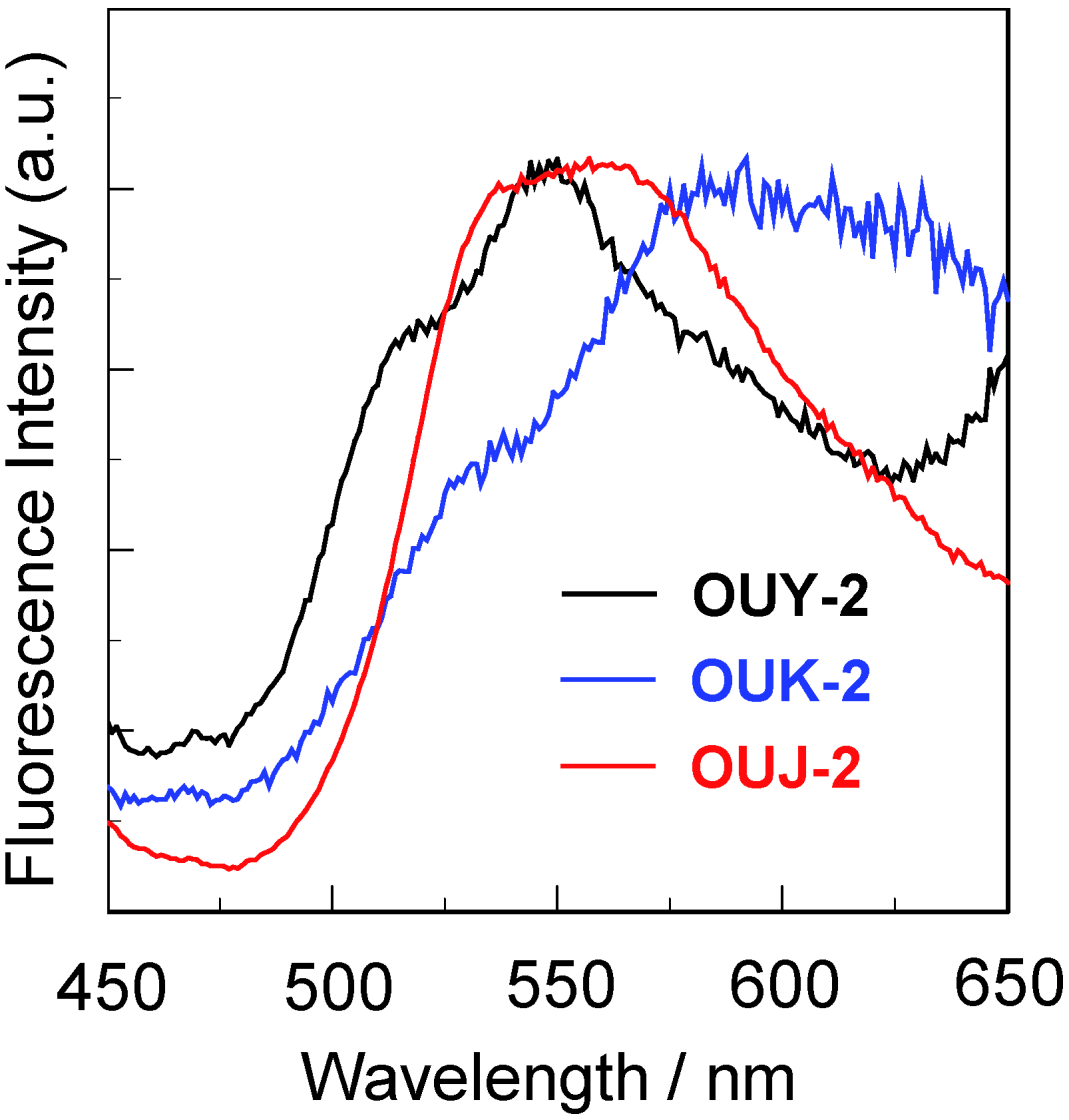
Fluorescence spectra of **OUY-2** (λ^ex^ = 370 nm), **OUK-2** (λ^ex^ = 370 nm) and **OUJ-2** (λ^ex^ = 380 nm) in the solid state.

In order to investigate the solid-state photophysical properties of **OUY**-**2**, **OUK-2** and **OUJ-2**, we have measured the solid-state fluorescence spectra of the solids (Figure 4). The λ_max,fl_ of the as-recrystallized dyes appears at 550 nm for **OUY**-**2**, 592 nm for **OUK**-**2**, and 557 nm for **OUJ**-**2**, which showed a significant bathochromic shift by 97 nm, 112 nm, and 48 nm, respectively, compared with those in toluene. The solid-state Φ_f_ value is below 0.02 for **OUY**-**2** and **OUK-2** and 0.09 for **OUJ-2**, which are much lower than those in toluene. It is well known that D–π–A fluorescent dyes show bathochromic shifts of the λ_max,fl_ and lower Φ_f_ values by changing from the solution state to the solid state. This fact is attributed to the delocalization of excitons or excimers due to the formation of intermolecular π–π interactions [48–51] between the dye molecules in the solid state, although we could not prepare single crystals of **OUY**-**2**, **OUK-2** and **OUJ-2** for the X-ray structural analysis.

### Electrochemical properties

**Figure 5:**
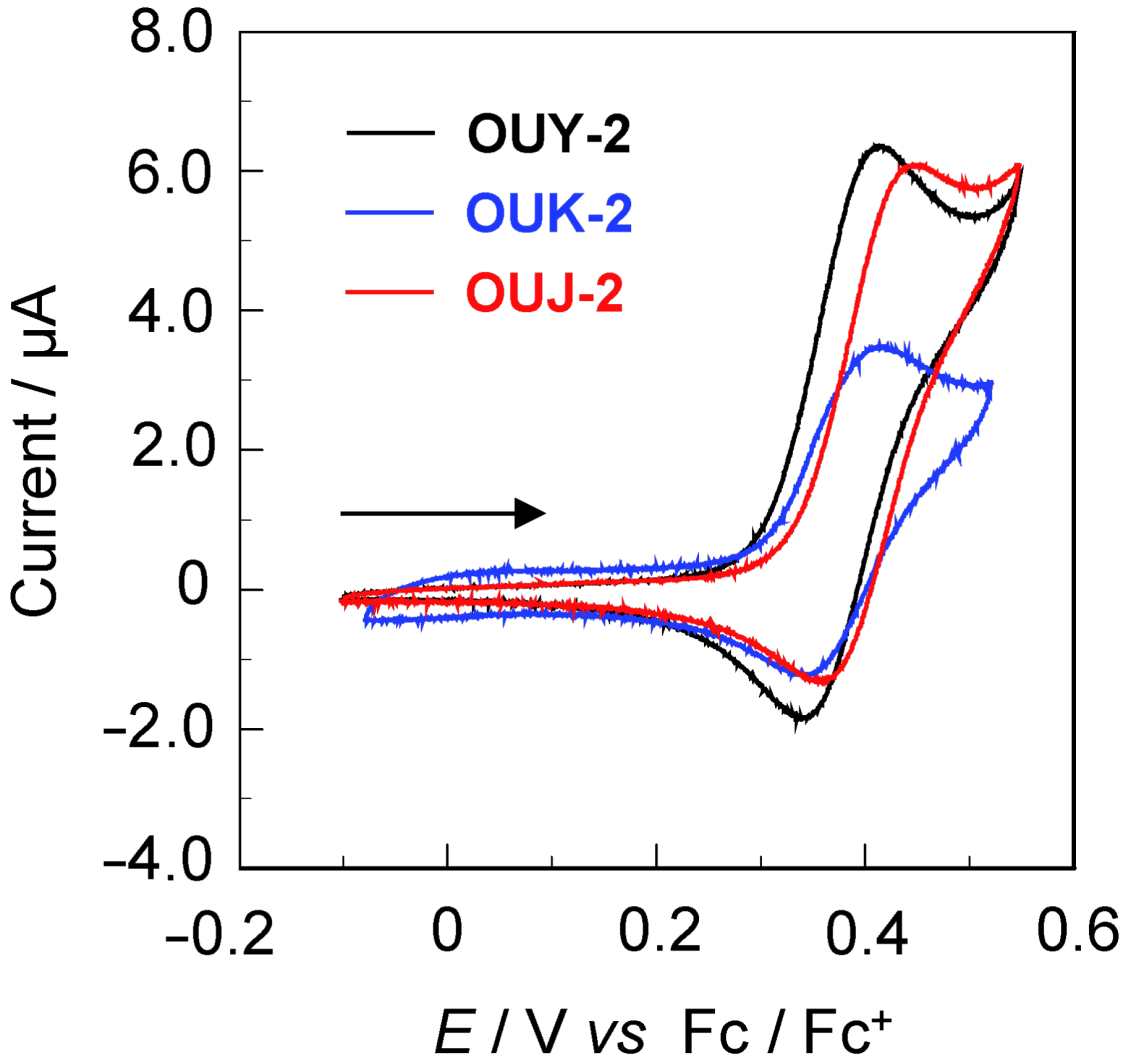
Cyclic voltammograms of **OUY-2**, **OUK-2** and **OUJ-2** in DMF containing 0.1 M Bu_4_NClO_4_. The arrow denotes the direction of the potential scan.

The electrochemical properties of **OUY**-**2**, **OUK-2** and **OUJ-2** were investigated by cyclic voltammetry (CV) in DMF containing 0.1 M tetrabutylammonium perchlorate (Bu_4_NClO_4_). The cyclic voltammograms of the three dyes are shown in Figure 5. The reversible oxidation waves (*E*_pa_^ox^) for the three dyes were observed at 0.42 V for **OUY**-**2** and **OUK-2** and 0.45 V for **OUJ-2**, vs ferrocene/ferrocenium (Fc/Fc^+^) (Table 2). The corresponding reduction waves (*E*_pc_^red^) appeared at 0.35 V for **OUY**-**2** and **OUK-2** and 0.36 V for **OUJ-2**, thus indicating that the three dyes undergo an electrochemically stable oxidation–reduction process. The HOMO energy level (− [*E*_1/2_^ox^ + 4.8] eV) versus the vacuum level was estimated from the half-wave potential for the oxidation (*E*_1/2_^ox^ = 0.39 V for **OUY**-**2** and **OUK-2** and 0.40 V for **OUJ-2**). Therefore, the HOMO energy level was −5.19 eV for **OUY**-**2** and **OUK-2** and −5.20 eV for **OUJ-2**, respectively. This fact indicates that the three dyes have comparable HOMO energy levels. The LUMO energy level versus the vacuum level was evaluated from the *E*_1/2_^ox^ and an intersection of photoabsorption and fluorescence spectra (449 nm; 2.76 eV for **OUY**-**2**, 481 nm; 2.58 eV for **OUK-2**, 506 nm; 2.45 eV for **OUJ-2**) in DMF. Consequently, the LUMO energy level was obtained through equation = [HOMO + *E*_0–0_] eV, where *E*_0–0_ transition energy is the intersection of the photoabsorption and fluorescence spectra corresponding to the optical energy gap between the HOMO and the LUMO. Thus, the LUMO energy level versus the vacuum level lowers in the order of **OUY-2** (−2.43 eV) > **OUK**-**2** (−2.61 eV) > **OUJ**-**2** (−2.75 eV). This result demonstrates that an increase of the electron-withdrawing ability of the azine ring lowers the LUMO energy level of the (D–π–)_2_A fluorescent dyes. Consequently, the fact revealed that the bathochromic shift of the ICT-based photoabsorption band in the order of **OUY-2** < **OUK-2** < **OUJ-2** is attributed to the stabilization of the LUMO energy level due to the increase in the electron-withdrawing ability of the azine ring in the order of pyridyl < pyrazyl < triazyl, resulting in a decrease in the energy gap between the HOMO and the LUMO.

**Table 2:** Electrochemical data and HOMO and LUMO energy level of **OUY-2, OUK-2** and **OUJ-2**.

Dye	*E*_pa_^ox^ [V]^a^	*E*_pc_^red^ [V]^a^	*E*_1/2_^ox^ [V]^a^	HOMO [eV]^b^	LUMO [eV]^c^	*E*_0–0_ [eV]^d^

**OUY-2**	0.42	0.35	0.39	−5.19	−2.43	2.76 eV
**OUK-2**	0.42	0.35	0.39	−5.19	−2.61	2.58 eV
**OUJ-2**	0.45	0.36	0.40	−5.20	−2.75	2.45 eV

^a^The anodic peak (*E*_pa_^ox^), the cathodic peak (*E*_pc_^red^) and the half-wave (*E*_1/2_^ox^) potentials for oxidation vs Fc/Fc^+^ were recorded in DMF/Bu_4_NClO_4_ (0.1 M) solution; ^b^the HOMO energy level (−[*E*^ox^_1/2_ + 4.8] eV) versus the vacuum level was evaluated from the *E*_1/2_^ox^ for oxidation; ^c^the LUMO energy level versus the vacuum level was evaluated from the HOMO and the optical energy gap (*E*_0–0_), that is, the LUMO energy level was obtained through equation = [HOMO + *E*_0–0_] eV; ^d^the optical energy gap (*E*_0–0_) was determined from the intersection of the photoabsorption and fluorescence spectra in DMF.

### Theoretical calculations

**Figure 6:**
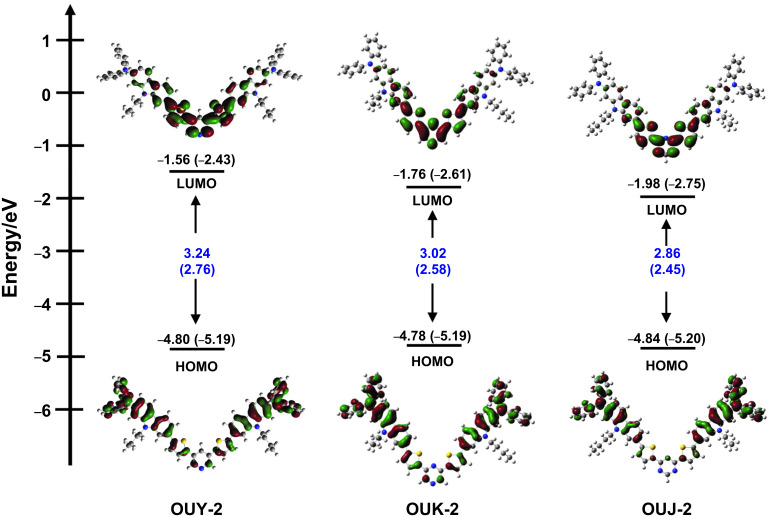
Energy level diagram, HOMO and LUMO of **OUY-2**, **OUK-2** and **OUJ-2**, derived from the DFT calculations at the B3LYP/6-31G(d,p) level of theory. Numbers in parentheses are the experimental values.

In order to examine the HOMO and LUMO distributions of **OUY**-**2**, **OUK-2** and **OUJ-2**, the molecular structures and the molecular orbitals of the three dyes were calculated using the DFT at the B3LYP/6-31G(d,p) level of theory [47]. The results of the DFT calculation for the three dyes indicated that the HOMO is mostly localized on the two (diphenylamino)carbazole moieties containing the thiophene ring and the LUMO is mostly localized on the thienylpyridine moiety for **OUY**-**2**, the thienylpyrazine moiety for **OUK-2** and the thienyltriazine moiety for **OUJ-2** (Figure 6). Accordingly, the DFT calculations reveal that the photoexcitation of **OUY**-**2**, **OUK-2** and **OUJ-2** induces the ICT from the two (diphenylamino)carbazole moieties to each azine ring. The HOMO energy level of the three dyes is remarkably similar to each other (−4.80 eV, −4.78 eV and −4.84 eV for **OUY**-**2**, **OUK-2** and **OUJ-2**, respectively), and the LUMO energy level is lowered in the order of **OUY-2** (−1.56 eV) > **OUK**-**2** (−1.76 eV) > **OUJ**-**2** (−1.98 eV), which are in good agreement with the experimental results from the photoabsorption and fluorescence spectral analyses (Figure 2) and the cyclic voltammetry (Figure 5). Thus, the experimental results and the DFT calculation strongly demonstrated that the bathochromic shift of the ICT-based photoabsorption band in the order of **OUY-2** < **OUK-2** < **OUJ-2** is attributed to a stabilization of the LUMO energy level due to the increase in the electron-withdrawing ability of the azine ring in the order of pyridyl < pyrazyl < triazyl.

## Conclusion

To gain insight into the photophysical and electrochemical properties of D–π–A fluorescent dyes with azine rings as electron-withdrawing groups, we have designed and synthesized a new type of (D–π–)_2_A fluorescent dyes **OUY**-**2**, **OUK-2** and **OUJ-2** with two (diphenylamino)carbazole thiophene units as the D (electron-donating group)–π (π-conjugated bridge) moiety and a pyridine, pyrazine or triazine ring as the electron-withdrawing group (electron-accepting group, A), and their photophysical and electrochemical properties were investigated. It was found that the intramolecular charge-transfer (ICT)-based photoabsorption and fluorescence bands of the three dyes appear at a longer wavelength region in the order of **OUY-2** < **OUK-2** < **OUJ-2**. This result is due to the increase in the electron-withdrawing ability of the azine ring in the order of pyridyl < pyrazyl < triazyl. Moreover, the (D–π–)_2_A fluorescent dyes showed a large bathochromic shift of the fluorescence maxima with increasing solvent polarity (i.e., positive fluorescence solvatochromism). The Lippert–Mataga plots revealed that the difference in the dipole moment of the dye between the excited state and the ground state increases in the order of **OUY-2** < **OUK-2** < **OUJ-2**. Thus, the fact explains our findings that **OUJ-2** shows large bathochromic shifts of fluorescence maxima in polar solvents, as well as the Stokes shift values for **OUJ-2** in polar solvents are much larger than those in nonpolar solvents. Cyclic voltammetry and DFT calculations demonstrated that the HOMO energy levels of the three dyes are remarkably similar, but the LUMO energy level is lowered in the order of **OUY-2** > **OUK-2** > **OUJ-2**, showing that increasing the electron-withdrawing ability of the azine ring lowers the LUMO energy level of the (D–π–)_2_A fluorescent dyes. Consequently, this work reveals that for the (D–π–)_2_A fluorescent dyes **OUY**-**2**, **OUK-2** and **OUJ-2**, the bathochromic shift of photoabsorption and fluorescence maxima and the lowering of the LUMO energy levels are dependent on the electron-withdrawing ability of the azine ring which increases in the order of **OUY-2** < **OUK-2** < **OUJ-2**.

## Experimental

### General methods

Melting points were measured with a Yanaco micro melting point apparatus MP model. FTIR spectra were recorded on a Shimadzu IRAffinity-1 spectrometer by ATR method. High-resolution mass spectra were acquired on a Thermo Fisher Scientific LTQ Orbitrap XL. ^1^H NMR and ^13^C NMR spectra were recorded on a Varian-400 (400 MHz) FT NMR spectrometer. Photoabsorption spectra were measured with a Hitachi U-2910 spectrophotometer, and fluorescence spectra were measured with a Horiba FluoroMax-4 spectrofluorometer. The fluorescence quantum yields in solution were determined by a Horiba FluoroMax-4 spectrofluorometer by using a calibrated integrating sphere system. Cyclic voltammetry (CV) curves were recorded in DMF/Bu_4_NClO_4_ (0.1 M) solution with a three-electrode system consisting of Ag/Ag^+^ as reference electrode, a Pt plate as working electrode, and Pt wire as counter electrode by using an electrochemical measurement system HZ-7000 (Hokuto Denko).

### Synthesis

#### General synthetic procedure of (D–π–)_2_A fluorescent dyes **OUY-2**, **OUK-2** and **OUJ-2**

**OUY-2** [2], **OUK-2** [3] and **OUJ-2** were prepared by Stille coupling of stannyl compound **1** [3] with 3,5-dibromopyridine, 2,6-diiodopyrazine, and 2,4-dichloro-1,3,5-triazine, respectively, by using Pd(PPh_3_)_4_ as a catalyst in toluene at 110 °C under an argon atmosphere (Scheme 1).

**Synthesis of OYJ-2:** A solution of **1** [3] (0.60 g, 0.95 mmol), 2,4-dichloro-1,3,5-triazine (0.071 g, 0.48 mmol), and Pd(PPh_3_)_4_ (0.18 g, 0.16 mmol) in toluene (10 mL) was stirred for 48 h at 110 °C under an argon atmosphere. After concentrating under reduced pressure, the resulting residue was dissolved in dichloromethane and washed with water. The dichloromethane extract was evaporated under reduced pressure. The residue was chromatographed on silica gel (ethyl acetate/dichloromethane 1:4 as eluent) to give **OUJ**-**2** (0.38 g, yield 70%) as yellow solid; mp 267–269 °C; IR (ATR) ν*~*: 1594, 1548, 1491 cm^−1^; ^1^H NMR (400 MHz, CD_2_Cl_2_) δ 0.89 (t, *J* = 7.3 Hz, 6H), 1.29–1.35 (m, 4H), 1.75–1.83 (m, 4H), 4.22 (t, *J* = 7.1 Hz, 4H), 6.96 (dd, *J* = 1.8 and 8.4 Hz, 2H), 7.02–7.06 (m, 4H), 7.13–7.16 (m, 10H), 7.26–7.30 (m, 8H), 7.57 (d, *J* = 4.0 Hz, 2H), 7.60–7.63 (dd, *J* = 8.1 and 1.5 Hz, 2H), 7.72 (s, 2H), 7.95 (d, *J* = 8.4 Hz, 2H), 8.04 (d, *J* = 8.1 Hz, 2H), 8.27 (d, *J* = 4.0 Hz, 2H), 9.00 (s, 1H) ppm; ^13^C NMR (100 MHz, CD_2_Cl_2_) δ 14.04, 20.86, 31.47, 43.10, 105.17, 106.55, 117.52, 117.92, 118.47, 120.53, 121.40, 123.08, 123.94, 124.50, 124.79, 129.58, 130.62, 133.57, 139.41, 141.56, 142.95, 147.31, 148.55, 153.55, 167.62 ppm (one aromatic carbon signal was not observed due to overlapping resonances); HRMS–ESI (*m*/*z*): [M + H] ^+^ calcd. for C_67_H_56_N_7_S_2_, 1022.40331; found, 1022.40344.

## Supporting Information

File 1 ^1^H and ^13^C NMR spectra of **OUJ-2**.
