# Supplementary material for: Synthesis, photophysical and electrochemical properties of pyridine, pyrazine and triazine-based (D–π–)2A fluorescent dyes
Source: Beilstein J Org Chem. 2019 Jul 22;15:1712–21. doi: 10.3762/bjoc.15.167 (PMC6664397; doi:10.3762/bjoc.15.167)
Supplement: File 1 — 1H and 13C NMR spectra of OUJ-2. [file Beilstein_J_Org_Chem-15-1712-s001.pdf]

BEILSTEIN JOURNAL OF ORGANIC CHEMISTRY

Supporting Information

for

Synthesis, photophysical and electrochemical properties of pyridine, pyrazine and triazine-based (D–π–)$_2$A fluorescent dyes

Keiichi Imato, Toshiaki Enoki, Koji Uenaka and Yousuke Ooyama

Beilstein J. Org. Chem. **2019,** *15,* 1712–1721. doi:10.3762/bjoc.15.167

^{1}H and ^{13}C NMR spectra of OUJ-2

License and Terms: This is a supporting information file under the terms of the Creative Commons Attribution License (http://creativecommons.org/licenses/by/4.0). Please note that the reuse, redistribution and reproduction in particular requires that the authors and source are credited. The license is subject to the *Beilstein Journal of Organic Chemistry* terms and conditions: (https://www.beilstein-journals.org/bjoc)

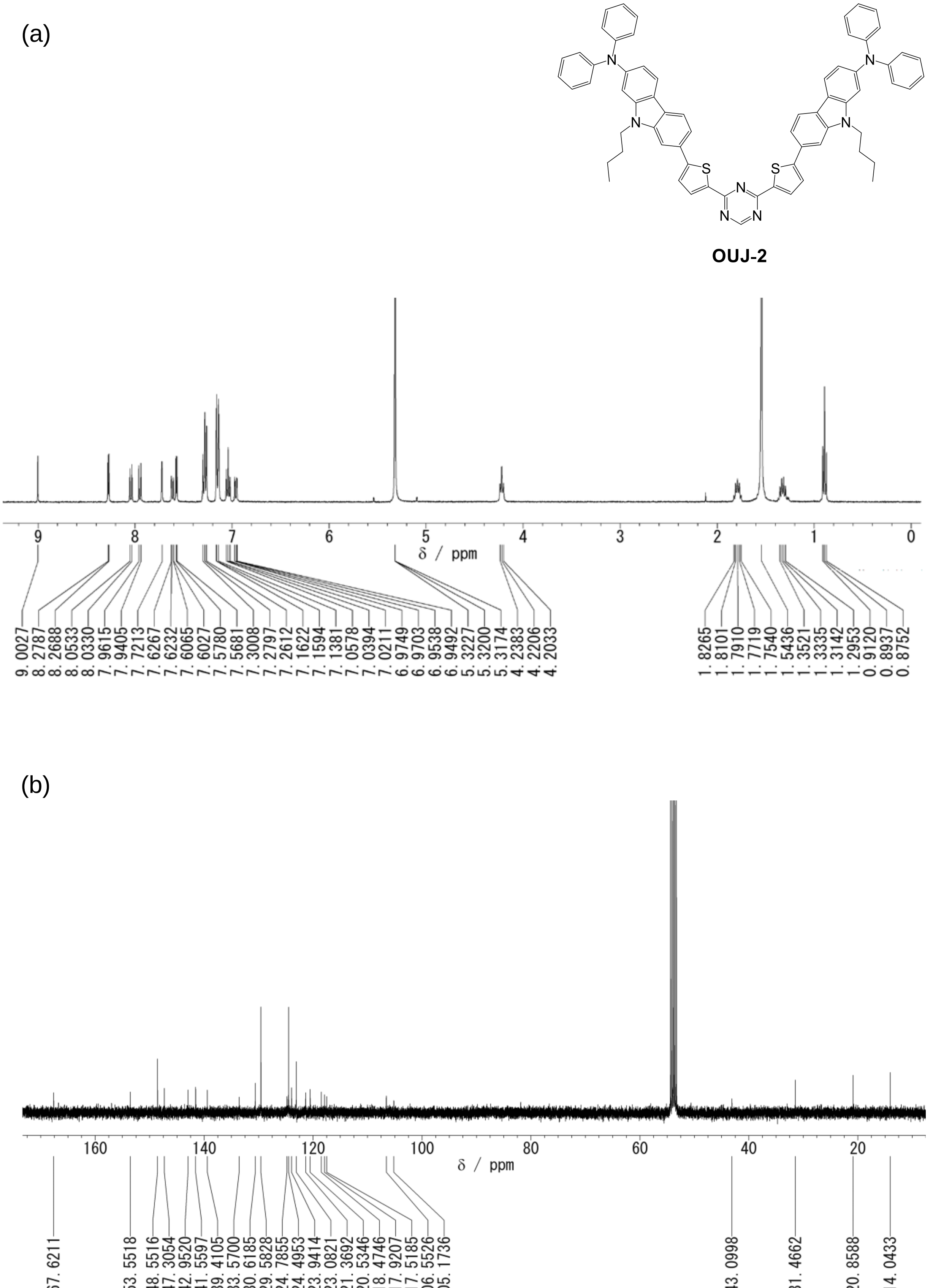

Figure S1: (a) ^{1}H HMR (400 MHz) and (b) ^{13}C HMR (100 MHz) spectra of **OUJ-2** in CD_2Cl_2.
